# Improved genomic island predictions with IslandPath-DIMOB

**DOI:** 10.1093/bioinformatics/bty095

**Published:** 2018-02-23

**Authors:** Claire Bertelli, Fiona S L Brinkman

**Affiliations:** Department of Molecular Biology and Biochemistry, Simon Fraser University, Burnaby, Canada

## Abstract

**Motivation:**

Genomic islands (GIs) are clusters of genes of probable horizontal origin that play a major role in bacterial and archaeal genome evolution and microbial adaptability. They are of high medical and industrial interest, due to their enrichment in virulence factors, some antimicrobial resistance genes and adaptive metabolic pathways. The development of more sensitive but precise prediction tools, using either sequence composition-based methods or comparative genomics, is needed as large-scale analyses of microbial genomes increase.

**Results:**

IslandPath-DIMOB, a leading GI prediction tool in the IslandViewer webserver, has now been significantly improved by modifying both the decision algorithm to determine sequence composition biases, and the underlying database of HMM profiles for associated mobility genes. The accuracy of IslandPath-DIMOB and other major software has been assessed using a reference GI dataset predicted by comparative genomics, plus a manually curated dataset from literature review. Compared to the previous version (v0.2.0), this IslandPath-DIMOB v1.0.0 achieves 11.7% and 5.3% increase in recall and precision, respectively. IslandPath-DIMOB has the highest Matthews correlation coefficient among individual prediction methods tested, combining one of the highest recall measures (46.9%) at high precision (87.4%). The only method with higher recall had notably lower precision (55.1%). This new IslandPath-DIMOB v1.0.0 will facilitate more accurate studies of GIs, including their key roles in microbial adaptability of medical, environmental and industrial interest.

**Availability and implementation:**

IslandPath-DIMOB v1.0.0 is freely available through the IslandViewer webserver {{http://www.pathogenomics.sfu.ca/islandviewer/}} and as standalone software {{https://github.com/brinkmanlab/islandpath/}} under the GNU-GPLv3.

**Supplementary information:**

Supplementary data are available at *Bioinformatics* online.

## 1 Introduction

Horizontal gene transfer (HGT) is widely recognized as a major force that drives microbial genome evolution. HGT enables bacteria and archaea to acquire foreign genetic material using various mechanisms, primarily conjugation, transduction and transformation (Dobrindt *et al.*, 2004; Soucy *et al.*, 2015). HGT disseminates beneficial, neutral and nearly neutral genes in integration hotspots, often tRNAs and tmRNAs, or interspersed within the core genome (Rodriguez-Valera *et al.*, 2016). The core genome of bacteria generally only represents on average 50% of the total genome size (Rodriguez-Valera *et al.*, 2016). Clusters of genes known or predicted to be acquired by HGT are called genomic islands (GIs), and were historically classified into different subtypes depending on the functions they encoded: symbiotic islands, metabolic islands, fitness islands, pathogenicity islands or antibiotic resistance islands (Hacker *et al.*, 1990; Juhas *et al.*, 2007, 2009; Sullivan and Ronson, 1998). GIs were shown to disproportionally encode virulence factors (Ho Sui *et al.*, 2009) and to be an important source of novel genes (Hsiao *et al.*, 2005), antimicrobial resistance genes (von Wintersdorff *et al.*, 2016), and metabolic genes (Juhas *et al.*, 2009) that essentially provide a selective or adaptive advantage to their hosts. The mobilization and transfer of GIs among a bacterial population is promoted by mobile selfish elements such as integrases, transposases and insertion sequences (Soucy *et al.*, 2015).

Given the growing appreciation of the importance of GIs for both environmental microorganisms and pathogens, the past decade has seen the development of numerous GI visualization and prediction software that are traditionally divided into sequence composition-based methods or comparative genomics methods. Most composition-based methods rely on the identification of nucleotide composition biases, including both measures of heterogeneity and homogeneity, in some cases combined with the identification of GI hallmarks (mobility and phage-related genes, tRNAs, direct repeats) (Che *et al.*, 2014; Langille *et al.*, 2010; Lu and Leong, 2016). Although most HGTs occur between closely related organisms and are difficult to identify owing to the similarity between the donor and the recipient genome, composition-based methods perform well in identifying recent transfers between more distantly related microorganisms or mobile element gene pools. They have an advantage of not requiring related genomes for comparison to detect GI regions. However, now that more genomes suitable for comparison exist, methods based on comparative genomics such as IslandPick (Langille *et al.*, 2008), MOSAIC (Chiapello *et al.*, 2005) and Gipsy (Soares *et al.*, 2016) may now be used further. The most widely used comparative genomics tool, IslandPick, uses comparative genomics with monophyletic groups of strains to identify regions unique to closely related bacteria that are absent from more distantly related bacteria. However, these latter methods still do require that enough genome sequences of sufficiently related isolates are available, and cannot detect islands shared between genomes compared, which may still be of medical and environmental interest.

Among standalone tools based on sequence composition analysis, SIGI-HMM (Merkl, 2004) and IslandPath-DIMOB (Hsiao *et al.*, 2003) were previously shown to have the highest overall precision and accuracy to predict GIs based on a test set built by comparative genomics using IslandPick (Langille *et al.*, 2008). SIGI-HMM predictions are based on the detection of biased codon usage using a hidden Markov model for each gene (Waack *et al.*, 2006), whereas IslandPath-DIMOB identifies genomic regions with biased dinucleotide composition encoding at least eight genes, of which at least one is a mobility gene (transposase, integrase, etc.), reflecting previously published and accepted criteria (Langille *et al.*, 2008, 2010). Within the past year, several new window-based methods identifying biases in sequence composition have been developed, often claiming higher recall and precision than previously existing tools. MSGIP (de Brito *et al.*, 2016), a standalone tool based on a mean shift clustering algorithm using single nucleotide composition, was shown to successfully identify known GIs from six bacterial genomes. Zisland Explorer (Wei *et al.*, 2016) uses cumulative GC profile to identify regions harboring a heterogeneous GC composition compared to the core genome and a homogenous GC composition within the region. When assessed using a dataset of 11 genomes using curated GIs from the literature, or GIs from a comparative genomics approach, Zisland Explorer exhibited at least 10% higher recall than IslandPath-DIMOB v0.2.0 and SIGI-HMM leading to a 4.9% higher overall accuracy. Finally, MTGIpick (Dai *et al.*, 2016) uses a *t*-test to identify regions with biased tetranucleotide composition before refining putative GI boundaries with a GC segmentation method and the Markovian Jensen–Shannon divergence (MJSD), as proposed by Arvey et al (2009). While these new methods provide essential novel approaches to GI prediction, their accuracy was determined using different datasets preventing the comparison of their respective capabilities.

Although it was developed over a decade ago, IslandPath-DIMOB has remained one of the methods with highest recall and overall accuracy. To integrate the latest knowledge gathered from comparative genomics and refine the prediction of GIs, we have developed a new version of IslandPath-DIMOB, part of the IslandViewer suite of GI analysis tools (Bertelli *et al.*, 2017), by implementing (i) a better score of dinucleotide bias to increase sensitivity, (ii) new extended HMM profiles to search for mobility genes, (iii) a better handling of pseudogenes and (iv) the concept of regions of GIs by considering closely positioned GIs as a single region. Furthermore, to provide a standardized base for GI prediction tool comparison, we created an improved GI dataset (derived from Langille *et al.*, 2008), identified by comparative genomics in 104 genomes, as well as a set of GIs retrieved from literature review. The prediction accuracy of the most popular and most recent tools, including IslandPath DIMOB v1.0.0, was then assessed using both datasets, allowing all of these methods to be compared with each other.

## 2 Materials and methods

### 2.1 IslandPath-DIMOB v1.0.0 implementation

IslandPath-DIMOB predicts GIs based on both the detection of dinucleotide bias in eight genes or more, and the identification of a mobility gene in the same region (Hsiao *et al.*, 2005, 2003). The detection of dinucleotide bias is much more sensitive than conventional GC content analysis to identify putative GIs. Furthermore, the required presence of a mobility gene reduces false positive predictions such as highly expressed genes that also exhibit abnormal sequence composition. To improve GI predictions, key modifications were implemented in the algorithm that identifies regions harboring dinucleotide biases, and the identification of mobility genes.

#### 2.1.1 Algorithmic modifications

The algorithm to score biases in dinucleotide composition has been preserved, as previously described (Hsiao *et al.*, 2003). Briefly, a score *S* is calculated as the average absolute dinucleotide relative abundance difference using a sliding window of six consecutive genes, shifting by one gene at a time. To improve the global sensitivity of the method and avoid decreased sensitivity in genomes with large numbers of recent gene acquisition, the median score *MedS* rather than the mean is calculated over all the windows in the genome. All regions scoring higher than the *MedS *+* *2*SD(*S*) are entirely retained, whereas in regions scoring between *MedS *+* *SD(*S*) and *MedS *+* *2*SD(*S*) only the first half of the genes are retained for further steps. Regions with dinucleotide biases spanning eight genes or more are conserved. Decreasing the required region length between three and five genes significantly increases the recall but also decreases the precision to a lesser extent. However, to conserve the desired high precision of IslandPath-DIMOB, the requirement for at least eight consecutive genes harboring dinucleotide biases was kept. They are merged into one single region if separated by five genes or less in order to correct for the observed tendency of IslandPath-DIMOB to split large GIs into small predicted regions.

#### 2.1.2 Mobility gene identification

The identification of mobility genes is performed in two independent parallel steps: Based on the identification of (i) known Pfam (Finn *et al.*, 2016) domains in proteins and (ii) keywords in protein functional annotation. First, a manually curated database of recent Pfam domains associated with integrase, transposase, resolvase and recombinase was created to replace the old outdated database of mobility genes. Domains specific to eukaryotic proteins were discarded. Domains of unknown function (DUF) shown to be associated to mobility genes were conserved as these could facilitate the identification of new GIs in novel poorly studied organisms that are not represented in the reference dataset (see below). To avoid false positives, a lower e-value cutoff, 10^−7^, was used for domain identification by HMMER3 v3.1b2 (Eddy, 2011). Second, an improved list of keywords was integrated, to search for mobility genes in the coding sequence product annotation, including IstB, insertion element, recombinase, insertion sequence, resolvase, integrase, phage, transposase, transposon, transposable element and excisionase.

#### 2.1.3 Ease of use and standalone version

To facilitate and standardize the use of IslandPath-DIMOB v1.0.0 as a standalone software, a new perl module has been added to accept as input a single genbank or embl flatfile. Other file formats previously required (ffn, faa and ptt) are now generated automatically and temporarily from the input flatfile. Furthermore, the software has been adapted to correctly process genomes with redundant protein gi identifiers, given that identical proteins in a genome now harbor identical gi and protein accession numbers since the change in NCBI’s RefSeq annotation policy in 2014. The standalone version of IslandPath-DIMOB v1.0.0 is freely available at https://github.com/brinkmanlab/islandpath/releases/. Furthermore, a version of IslandPath-DIMOB v1.0.0 is now integrated in the webserver IslandViewer 4: http://www.pathogenomics.sfu.ca/islandviewer/ (Bertelli *et al.*, 2017). Finally, to facilitate the use of IslandPath-DIMOB in cloud environment and remove the need to install the software and its dependencies, a docker image is also available at https://hub.docker.com/r/brinkmanlab/islandpath/.

### 2.2 Reference dataset of genomic islands

#### 2.2.1 Old genome files

To assess the improvement of IslandPath-DIMOB v1.0.0 compared to IslandPath-DIMOB v0.2.0 (Hsiao *et al.*, 2005), genome files from RefSeq were used as available before July 2014, that is before NCBI’s reannotation initiative. Indeed, the reannotation initiative introduced non-redundant protein accession numbers that are not supported by the old IslandPath-DIMOB v0.2.0 version, preventing us from assessing its accuracy with newer files.

#### 2.2.2 New genome files

To assess the accuracy of IslandPath-DIMOB v1.0.0 and other recent GI prediction methods, genome files were retrieved from RefSeq by MicrobeDBv2 (Langille *et al.*, 2012) on Feb 09, 2017. Accession and version numbers, and thus genome sequence, for the old genome files and the new genome files are strictly identical and available along with the organism name in Supplementary Table S1. The annotation of genomes may differ between the old and the new genome files.

#### 2.2.3 Comparative genomics-based dataset (C-dataset)

To obtain a reliable and independent reference dataset of GIs identified by methods not relying on nucleotide bias/compositions, we first retrieved the dataset obtained by using the comparative genomics approach of IslandPick (Langille *et al.*, 2008). Among the 118 genomes in the original dataset, 14 were discarded because new genome sequence versions were released since the initial analyses, which could have led to possible inaccuracies in genomic coordinates for subsequent analyses. We then created an improved dataset of GIs, benefitting from the considerable increase in genome sequences available for comparative genomics approaches since 2008. The original dataset was combined with current GI predictions by IslandPick available as pre-computed results in IslandViewer 3 (Dhillon *et al.*, 2015) and IslandViewer 4 (Bertelli *et al.*, 2017) for the same 104 genome sequences to form a reference positive dataset (Supplementary Table S1). Each of the 104 genome in the reference positive dataset harbored between 1 and 77 GIs larger than 4 kb, for a total of 1845 GIs encompassing over 21 Mbp.

As the result of new genomes being available for the IslandPick comparative approach, the negative dataset of core genomic regions inferred by Langille *et al.* (2008) was adapted in four Burkholderia genomes (NC_008390.1, NC_008061.1, NC_010515.1, NC_010084.1) to remove a few regions now predicted as horizontally transferred by IslandPick (Supplementary Table S2). The negative dataset comprises 3266 regions, ranging in size between 7 and 82 kb, for a total of over 45 Mbp. These core regions are conserved in each reference genome and its related genomes selected for comparison by IslandPick at varying genomic distance (Bertelli *et al.*, 2017; Dhillon *et al.*, 2015; Langille *et al.*, 2008).

#### 2.2.4 Curated literature-based dataset (L-dataset)

To evaluate the ability of different software to predict well-defined GIs obtained by other groups using independent methods, a literature dataset was created by reviewing articles describing GIs in some well characterized organisms. The literature dataset from Langille *et al.* (Langille *et al.*, 2008) was used and extended to include six genomes: *Escherichia coli* O157: H7 str. Sakai (NC_002695.1)*, Escherichia coli CFT073* (NC_004431.1), *Salmonella enterica subsp. enterica serovar Typhi* str. CT18 (NC_003198.1), *Streptococcus pyogenes* str. MGAS315 (NC_004070.1), *Vibrio parahaemolyticus* RIMD 2210633 (NC_004603.1) and *Staphylococcus aureus* str. MW2 (NC_003923.1). Two genomes from the literature dataset of Langille *et al.* were discarded due to changes in accession version number (NC_002655.2: *Escherichia coli* O157: H7 EDL933, NC_003197.1: *Salmonella typhimurium* LT2) that could have impacted the accuracy of GI coordinates. Overall, the literature dataset comprises 80 GIs ranging in size from 3 to 133 kb, encompassing over 3 Mbp in total (Supplementary Table S3).

Both the C-dataset and the L-dataset are available in tabular format as Supplementary table in this contribution. Tabular as well as fasta formats are also available on IslandViewer 4 website (http://www.pathogenomics.sfu.ca/islandviewer/download/).

### 2.3 Software accuracy assessment

The most recently published tools Zisland Explorer (Wei *et al.*, 2016), MTGIpick (Dai *et al.*, 2016), MSGIP (de Brito *et al.*, 2016) as well as older highly accurate tools SIGI-HMM (Waack *et al.*, 2006), IslandPath-DIMOB v0.2.0 (Hsiao *et al.*, 2005) and Islander (Hudson *et al.*, 2015) were used to predict GIs on the reference dataset of 104 genomes (Supplementary Table S1). Each software or webserver was run using default parameters. For SIGI-HMM, any region with at least two consecutive genes identified as putative horizontal transfers was counted as a predicted GI. MTGIpick was run without the boundary refinement option as the selection of the option would result in an error message on the webserver (and it is not set as a default parameter).

Since accuracy metrics vary largely depending on the bacterial genome considered, we calculated here the following metrics per genome for each tool:

$$Recall=TPR=\frac{TP}{TP+FN}$$

$$Precision=PPV=\frac{TP}{TP+FP}$$

$$Overall\;accuracy=ACC=\frac{TP+TN}{TP+FP+FN+TN}$$

$$F1\;score=F1=\frac{2TP}{2TP+FP+FN}$$

$$MCC=\frac{TPxTN-FPxFN}{\sqrt{\left(TP+FP)(TP+FN)(TN+FP)(TN+FN\right)}}$$

Where TP, FP, FN and TN are true positives, false positives, false negatives and true negatives, respectively. All bases included in both the reference positive dataset and the predicted GIs were counted as true positives (TP) while bases only in the reference positive dataset were counted as false negatives (FN). The negative dataset of core genomic regions was used to assess the true negatives (TN) counted as all bases in the negative dataset that were not predicted as GIs, and false positives (FP) counted as all bases in the negative dataset that were predicted as GIs. In the edge case where TP and FP were equal to 0, the precision was counted as equal to 1, since the software is being conservative making no prediction. The Matthews correlation coefficient (MCC) was used as a measure of the correlation between the reference datasets and the observed predictions. MCC values vary between −1 and 1, with 1 representing a perfect prediction, 0 no better than random prediction and −1 a complete disagreement between the prediction and the reference dataset. MCC was considered as 0 when the denominator was equal to 0.

## 3 Results and Discussion

### 3.1 Validation of the new reference C-set

Since the first determination of the reference comparative dataset in 2008 (Langille *et al.*, 2008), numerous genomes have been released in public databases, enabling finer genome comparison. Therefore, pre-computed IslandPick predictions available in IslandViewer 3 (Dhillon *et al.*, 2015) as well as IslandViewer 4 (Bertelli *et al.*, 2017) were retrieved for the 104 genomes with matching accession and version numbers. Since IslandPick predictions depend on the genomes selected for comparison, the Langille’s reference dataset, IslandPick predictions in IslandViewer 3 and IslandPick predictions in IslandViewer 4 only partially overlap. Indeed, only between 3 and 6 genomes at varying phylogenetic distance are used for comparison in IslandViewer 3 and 4, whereas more extensive comparison were performed by Langille *et al.* to build the original reference dataset. For example, IslandPick predictions from IslandViewer 3 and IslandViewer 4 only include, respectively, 28% and 25% of the bases from the Langille’s dataset, whereas the Langille’s dataset includes 45% and 57% of the bases in IslandPick GI dataset of IslandViewer 3 and IslandViewer 4, respectively. It is expected that the first reference dataset contains more predicted GIs given the more extensive comparison performed by Langille et al. (Langille *et al.*, 2008). The three datasets were combined to form an updated reference positive dataset (Supplementary Table S1) that better represents the GIs encoded in the 104 reference genomes for the evaluation of GI prediction tools.

Most importantly, none of the novel GIs predicted by IslandPick in IslandViewer 3 overlap regions of the previously established negative dataset containing the core genomes. IslandPick predictions from IslandViewer 4 showed that regions previously considered as core genome in only four *Burkholderia* genomes where in fact probably horizontally acquired, leading to the modification of the negative reference dataset (Supplementary Table S2). The overall very limited overlap confirms the accuracy of the past negative dataset and new IslandPick predictions. Moreover, we investigated the overlap between the C-dataset and the L-dataset in the six genomes common to both datasets (Table 1, “literature dataset” and Table 2, “comparative dataset”). The L-dataset comprising curated GIs encompasses over 89% of the bases in the C-dataset and does not overlap with the core genomic regions of the negative dataset (precision equals 1), thus helping to confirm the validity of this new reference dataset (Table 1). The C-set only partly covers the L-dataset (37%) (Table 2), which suggests that the C-dataset is an underestimate but representative sample of the GIs present in these genomes.

**Table 1.** Mean GI prediction accuracy assessed using the 104 genomes of the reference C-dataset and overlap with the literature dataset in six genomes

	Method	MCC	F-score	Accuracy	Precision	Recall
Old genome files^a^	IslandPath-DIMOB v1.0.0	0.51	0.55	0.77	0.88	0.46
	IslandPath-DIMOB v0.2.0	0.39	0.44	0.73	0.83	0.34
New genome files^b^	IslandViewer 4	0.70	0.78	0.89	0.90	0.73
	IslandPath-DIMOB v1.0.0	0.49	0.55	0.77	0.87	0.47
	SIGI-HMM	0.35	0.37	0.73	0.92	0.26
	MTGIpick	0.32	0.56	0.70	0.55	0.68
	Zisland Explorer	0.20	0.23	0.69	0.85	0.18
	Islander	0.19	0.20	0.70	0.97	0.14
	MSGIP	0.15	0.20	0.68	0.87	0.16
Literature dataset	Literature	0.89	0.94	0.94	1	0.89

a Old genome files for the reference dataset as available in RefSeq before July 2014.

b New genome files for the reference dataset downloaded from RefSeq on February 9, 2017.

**Table 2.** Mean GI prediction accuracy and overlap with the C-dataset assessed using the reference L-dataset comprising six genomes

	Method	MCC	F-score	Accuracy	Precision	Recall
Multiple predictors	IslandViewer 4	0.64	0.75	0.79	0.998	0.62
Single predictors	IslandPath-DIMOB v1.0.0	0.54	0.67	0.72	0.979	0.52
	MTGIpick	0.50	0.78	0.75	0.82	0.74
	SIGI-HMM	0.36	0.42	0.60	0.998	0.29
	Islander	0.32	0.35	0.56	1	0.23
	MSGIP	0.31	0.44	0.62	0.95	0.35
	Zisland Explorer	0.18	0.26	0.52	0.83	0.17
Comparative dataset	C-dataset	0.43	0.51	0.65	1	0.37

### 3.2 Improvements of IslandPath-DIMOB

To assess the improvement of IslandPath-DIMOB, we compared the performance of the releases v0.2.0 and v1.0.0 on the updated reference comparative dataset (Supplementary Table S1) using the old genome files. IslandPath-DIMOB v0.2.0 showed a recall, precision and accuracy of 34.4%, 83.1% and 72.7%, respectively (Table 1). In addition to the different positive C-dataset used here, several other reasons explain the difference in the accuracy reported here for IslandPath-DIMOB v0.2.0 compared to the original assessment (Langille *et al.*, 2008) that showed a 35.6% recall, 85.8% precision and 86.2% accuracy. First, although the version of the genome accession number and thus the genome sequence itself is identical to that used in the analysis by Langille *et al.*, the genome annotation may have changed, which may impact the detection of mobility genes. Furthermore, the use of a newer version of HMMER3, instead of HMMER2, to identify mobility genes influences the results. Finally, to better reflect the variation in the ability to predict GIs in a variety of genomes, we calculated here the average of the recall, precision and accuracy for each genome rather than an overall value as performed previously (Langille *et al.*, 2008). The new IslandPath-DIMOB v1.0.0 has a recall, precision and accuracy of 46.1.8%, 88.4% and 77.1%, respectively. This represents a 11.7% increase in recall and a 5.3% increase in precision for IslandPath-DIMOB v1.0.0 compared to the previous release.

### 3.3 Assessment of prediction accuracy

To compare the performance of IslandPath-DIMOB v1.0.0 to the latest and the most accurate tools for GI prediction, we used both a reference dataset identified by comparative genomics (C-dataset) and a dataset from the literature (L-dataset).

#### 3.3.1 Prediction accuracy using the C-dataset

The software assessed vary greatly in their mean ability to predict GIs, generally with a tradeoff between recall and precision (Table 1). Islander has the highest precision (97.1%) but a low recall rate (14%), as can be expected since it only predicts canonic GIs inserted into tRNAs or tmRNAs, with both attachment sites conserved and encoding an integrase (Hudson *et al.*, 2015). SIGI-HMM follows closely with 91.9% precision, also with an intermediate recall of 26.4%. ZislandExplorer and MSGIP additionally have low recall (17.7% and 16.3%, respectively) with good precision above 85%. On the other hand, MTGIpick has the highest recall (67.5%) but a low precision (55.1%). IslandPath-DIMOB v1.0.0 has the second highest recall (46.9%) while retaining a high precision (87.4%). This is reflected in the overall accuracy as well as the F-score that factors both the recall and the precision. Among individual methods tested here, IslandPath-DIMOB v1.0.0 also obtains the highest Matthews correlation coefficient that is considered a balanced measure of the correlation between observed and predicted binary classification independent of class sizes. Due to its high recall, MTGIpick also obtains a high F-score but a markedly lower Matthews correlation coefficient. Finally, to show the value of using multiple GI predictors, we have included in the comparison IslandViewer 4, that integrates three methods (SIGI-HMM, IslandPath-DIMOB v1.0.0 and IslandPick that all have relatively high precision). It outperforms all the single-method predictors with an MCC score over 0.7 and a high recall (70.3%) while maintaining a high precision (>90%).

**Fig. 1.**
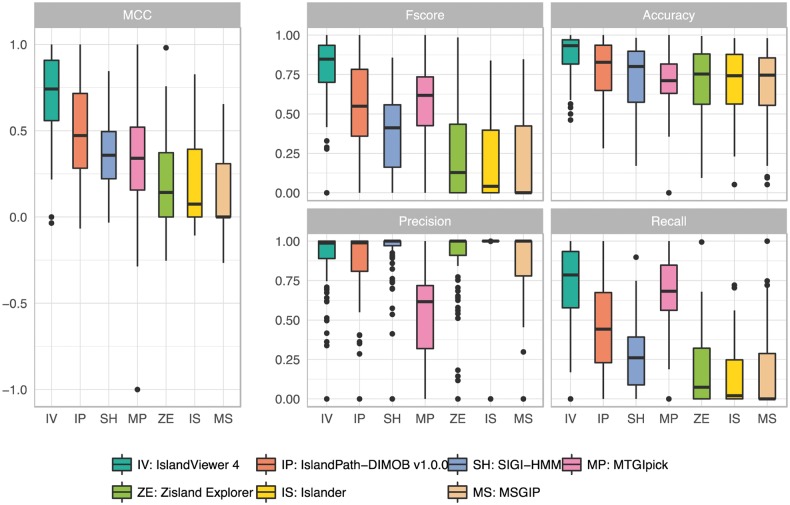
GI prediction accuracy of IslandPath-DIMOB and other individual methods compared with IslandViewer 4 composite method, based on the C-dataset (comparative genomics-based dataset). Prediction accuracy on the dataset of GIs identified by the comparative genomics approach IslandPick in 104 genomes. The boxplot shows the median, and the first and third quartiles as the lower and upper hinges. Outliers are indicated as black dots, if they exceed 1.5 times the interquartile range. IslandViewer 4 is a composite method, combining IslandPath-DIMOB, SIGI-HMM and IslandPick, whereas other tools are individual methods

Although widely used in the assessment of GI prediction tools, the mean of accuracy metrics might not represent well the overall performance of a software. Indeed, our analysis shows that, in most cases, a broad distribution of values is obtained by each software for the different genomes in the reference dataset (Fig. 1). For example, the mean precision is highly influenced by some genomes with very low values. More robust to outliers, the median precision of Islander, SIGI-HMM, MSGIP and ZislandExplorer is 100%, but IslandPath-DIMOB v1.0.0 follows closely with a median precision of 99%. This shows that, in most genomes, the latter tools do not predict GIs in highly conserved genomic regions. Most importantly, the variation of metrics depending on the genome highlights the need for large datasets to benchmark GI prediction tools, as a small number of genomes can easily lead to biased accuracy metrics. Furthermore, the use of similar datasets such as that from Langille *et al.* (2008) to train or develop methods and test these methods may bias the assessment of GI prediction software. As for IslandPath-DIMOB, other methods also have incorporated such islands in their training dataset and are affected by this issue. Therefore, it is essential to also assess GIs using an independent dataset such as well characterized GIs from literature reviews.

#### 3.3.2 Prediction accuracy using the L-dataset

GI predictions were also compared to GIs in six genomes that were precisely described in the literature. Most individual GI prediction tools show similar recall when using the L-dataset and the C-dataset. However, due to the small number of genomes in the L-dataset, the assessment is much more sensitive to the set of genomes used. In fact, most methods, except Zisland Explorer show a higher recall (between +3% for SIGI-HMM and +13% for MSGIP) than the average presented in Table 1 in these six genomes when assessed against the C-dataset. Three methods—Islander, SIGI-HMM and IslandPath-DIMOB—see a decrease in recall between −1% and −6% with the assessment against the L-dataset for these six genomes, and three methods—MTGIpick, Zisland Explorer and MSGIP—show an increase in recall of 0.5%, 3.3% and 5.4%, respectively. Also, large variations can be observed in the precision of the GI prediction tools. Since the negative dataset and thus the number of false positives remains identical in the C-dataset and L-dataset comparisons, the improvement in precision is due to the increased recall of most prediction methods as well as the limited number of genomes represented in the L-dataset. As mentioned above, small number of genomes might yield incorrect mean values due to a biased genome representation, thus stressing the importance to develop larger standardized dataset with manually curated GIs from the literature, or other analyses, to accurately assess GI predictors.

The recall of IslandViewer 4 is significantly decreased (to 61.9%) by the accuracy assessment using the L-dataset (Table 2). This is expected given that IslandPick predictions from IslandViewer 4 were all included in the reference C-dataset, thereby artificially increasing the recall of IslandViewer 4 in the C-dataset accuracy assessment. The true recall of IslandViewer 4 is therefore likely more correct in the case of the L-dataset. Nevertheless, IslandViewer 4 exhibits high recall and precision leading to the highest MCC score among predictors, which is considered a less-biased measure of correlation between the reference dataset and the predictions. It is followed by IslandPath-DIMOB v1.0.0, that still conserves the highest score among single prediction methods. Overall, these data confirm the very good accuracy of both the comparative genomics dataset, and this new IslandPath-DIMOB release, for the prediction of GIs in microbial genomes.

## 4 Conclusion

We report here a new version of IslandPath-DIMOB that significantly improves the identification of GIs in microbial genomes. We have improved and expanded the reference GI dataset (positive dataset) predicted by IslandPick that can be used for GI predictor evaluation, as well as a corrected negative dataset of non-GI regions derived from Langille *et al.* (2008). Given the large variation in accuracy observed for all the GI prediction tools among the different genomes within and across bacterial species, we strongly recommend the use of such large datasets to assess past and new GI prediction tools. Although the present dataset is not optimal because it only partly covers GIs based on curated literature review, it represents a good resource and a standardized reference to benchmark GI prediction tools, similar to the past dataset which was used to compare predictive methods. The accuracy assessment has confirmed that IslandPath-DIMOB remains a method of choice compared to other tools, and now provides higher recall at high precision. Its use in combination with other methods, in particular in the IslandViewer 4 webserver (Bertelli *et al.*, 2017), provides researchers with highly improved GI predictions for both pre-computed genome analysis based on the collection of NCBI genomes, plus more custom bacterial and archaeal genome analysis submitted by users to such a webserver. The standalone IslandPath-DIMOB version will also be useful for users with large-scale/local analyses needs, and those wishing to implement this method in their own pipelines. In the era of whole genome sequencing for environmental strains with enhanced adaptability (Juhas *et al.*, 2009) as well as for pathogen outbreak investigation (Bertelli and Greub, 2013; Fricke and Rasko, 2014), such GI prediction methods will remain key in identifying important genomic regions that can encode metabolic genes, virulence factors or antimicrobial resistance genes of particular environmental and medical relevance.

## Funding

This work was primarily supported by a Swiss National Science Foundation fellowship (P300PA_164673) and a grant from the Société Académique Vaudoise to C.B., with some support by Genome Canada and Cystic Fibrosis Foundation Therapeutics.

*Conflict of Interest*: none declared.

## Supplementary Material

Supplementary Data
